# Research on the causal mechanism of a rock burst accident in a longwall roadway and its prevention measures

**DOI:** 10.1038/s41598-023-41769-z

**Published:** 2023-12-15

**Authors:** Ying Chen, Zikai Zhang, Chen Cao, Shiji Bao, Shuai Wang, Guangyuan Xu

**Affiliations:** College of Mining, Liaoning Technical University, Fuxin, 12300 China

**Keywords:** Civil engineering, Geodynamics

## Abstract

Based on the disaster characteristics and the geo-conditions at the scene, in this study, the occurrence mechanism of a serious rock burst accident that occurred in the Tangshan Coal Mine, China, was analysed. Ground stress measurements showed that the mine is in a high ground stress area dominated by horizontal tectonic stress around 33 MPa. Laboratory testing revealed that the coal was a hard seam of 8.3 MPa over bedded by a thick and hard roof stratum with uniaxial compressive strength of 66 MPa. The calculation results indicated that the accident occurred in the roof rebounding area. It is proposed that the hard roof and the hard coal seam formed a seesaw structure around the working face. The vertical pressure relief caused the rib coal mass to lose its clamping forces from the roof and floor and rush into the roadway, resulting in a rock burst accident. Based on the causality mechanism of the rock burst disaster developed in this study, pertinent coal bump prevention measures have been undertaken in practice. Large-diameter boreholes were drilled to eliminate the pivot effect of the seam. Roof blasting was undertaken to prevent the roof from forming a seesaw plank. To summarize, a new causality mechanism for rock burst in coal mines under hard roof and hard seam geo-conditions was developed.

## Introduction

Coal forms the world's richest fossil energy reserves. In 2021, fossil fuels accounted for 82% of energy usage worldwide, and global coal consumption increased by more than 6% compared with 2020^1^. Underground coal mining engineering is a key part of interacting with the Earth to establish a sustainable modern society.

With the depletion of shallow underground resources, the buried depth of underground coal mines increases at an average rate of 10–25 m per year. The current mining depth in China has reached 600–900 m underground; in the future, the coal mining depth is expected^2^ to reach 1.5–3.0 km. The geological conditions of deep underground engineering have deteriorated greatly. Under the conditions of high ground stress and high temperature, high-intensity mining engineering causes frequent disturbances in the surrounding rock, which induce geological dynamic disasters such as rock burst, coal bumps, coal and gas outburst^2,3^. In recent years, the amount and severity of bumped coal mines in China has rapidly increased with increasing mining depth^4^. At approximately 12:30 on August 2, 2019, a rock burst accident occurred at the F5009 longwall panel of the Tangshan Mining Branch of Kailuan (Group) Co., Ltd. (hereinafter referred to as the Tangshan Coal Mine), resulting in 7 deaths^5^.

The mechanism of rock burst-induced disasters and their occurrence is extremely complex. In early research, some theories were proposed to try to explain the causality mechanism of coal bumps, including strength theory, stiffness theory, energy theory, and impact propensity theory^6–9^. Subsequent research has improved these theories. Based on the characteristics of rock burst occurrence, Jiang et al.^10^ classified rock burst into three modes, namely, material instability, slip dislocation, and structural instability, and analysed the instability of coal–rock combination samples under compression. Brauner^11^ first proposed the clamping effect of a coal seam and believed that the rib coal was in a clamped state between the roof and floor plates. When the interfaces between the coal body and the surrounding rock reached an equilibrium state, the coal body may have burst out due to compression failure. Qi et al.^12^ conducted experimental research on coal rock frictional sliding and proposed a stick-slide model of rock burst, which suggested that rock burst was the process of instantaneous stick–slip instability of coal rock layers. The structure of bedded coal rock and the thin and weak interlayers between them were the main factors leading to rock burst.

Rock burst in underground coal mines normally occurs in mining roadways. According to 2510 recorded rock burst data points, 2178 events occurred in mining roadways, accounting for 86.8% of the total^13^. The Tangshan Coal Mine rock burst disaster also occurred in a roadway. Coal bumps occur in mining roadways and are often characterized by the overall intrusion of rib coal masses into the roadway, which is also the most severe and frequent coal bump event. Therefore, research on the occurrence mechanism and prevention technology of rock bursts occurring in mining roadways is significant and urgent for underground coal mining safety.

Rock bursts that occur in mining roadways is the result of the disruption of the equilibrium of the surrounding rock and coal system. Petukhov et al.^14^ believed that coal bodies under plastic deformation at the boundary of coal seams were subjected to high pressure and underwent impact deformation after overcoming frictional forces generated by the roof and floor; the stored elastic compression potential energy was converted to kinetic energy, causing rock burst. Casten and Fajklewicz^15^ believed that rock burst was a critical state of coal and rock mass under high stress, resulting in dynamic instability underground disturbances. In recent years, there has been some progress towards an understanding of rock burst theory. Pan et al.^16^ proposed the concept of a critical resistance zone in rock burst, which emphasized that the ratio of the Young’s modulus to post-failure gradient ($E/\lambda$) of the coal seam is the most influential parameter inducing a rock burst. Based on the stress and energy characteristics of rock burst, Cai et al.^17^ analysed the changes in the strain rate of coal samples using laboratory experimental methods, and they proposed a coal bump theory of dynamic load and static load superposition.

A hard roof is a typical geo-condition for the occurrence of rock burst and is mainly composed of sandstone or conglomerate^18,19^. The hard roof forms a large area of suspended roof behind the longwall working face, which induces high abutment pressure on the coal ahead of the working face^20^. The larger the suspended area of the hard roof is, the more energy it accumulates. When the roof fractures, a significant amount of elastic and gravitational potential energy is released, leading to the destruction, expansion, and outward movement of the coal body, forming a rock burst^21^. Regarding the roof and floor as elastic material, Lippmann^19^ stated that the coal seam between them was in an equilibrium state under an overburden load. When a small disturbance acted on the coal seam, the coal seam could slide with the roof, which caused coal seam impact. In subsequent research, based on elastic foundation theory, Ma et al.^22^ established a finite elastic beam model to analyse the impact of a hard roof on a rock burst occurring in a longwall mining roadway.

Numerical methods have been introduced into the mechanistic study of rock burst. Li^23^ established a numerical model based on the Yima coalfield, China, and determined the thickness of the hard roof and the distance from the coal seam on the effect of stress transfer during working face advance. Zhang et al.^24^ studied the stress distribution of a mining roadway under a coal pillar in the Huoluowan coal mine and determined the range of influence of the upper seam pillar. Liang et al.^25^ conducted numerical calculations on the 1359p working face of the Qianjiaying Coal Mine, and they analysed the stress and deformation characteristics of the roadway after underlayer mining of four working faces. Zhu et al.^26^ studied the coal mining subsequence of the Tongxin Coal Mine using FLAC3D; the stress distribution of the coal seam was obtained under different roof strengths using a simplified numerical model.

The prevention and control of geological disasters is of great significance for sustainable development. Based on the rock burst accident in the Tangshan Coal Mine, in this research, we used a numerical calculation method to analyse the effect and influence of abutment pressure on the movement of the roof in front of the working face, and we established a theoretical model of the rock burst mechanism in the mining roadway under hard-roof conditions. Consequently, relevant prevention measures were designed and implemented to provide guarantees for mining safety. This study provides a new numerical method for research on geological dynamic disasters and technical support for the sustainable development of deep resource excavation.

## Materials and methods

Numerical methods can be used to establish calculation models for different engineering backgrounds to obtain the stress distribution and deformation pattern of surrounding rock under specific circumstances and are an important research tool for case studies. In this study, FLAC3D was utilized to analyse the cause and process of the accident. To improve the accuracy of the calculation results, ground stress measurements were carried out in the accident area, the overlying strata structure was detected, and the strength of coal seams and overlying strata were also measured.

### Engineering background

**Figure 1:**
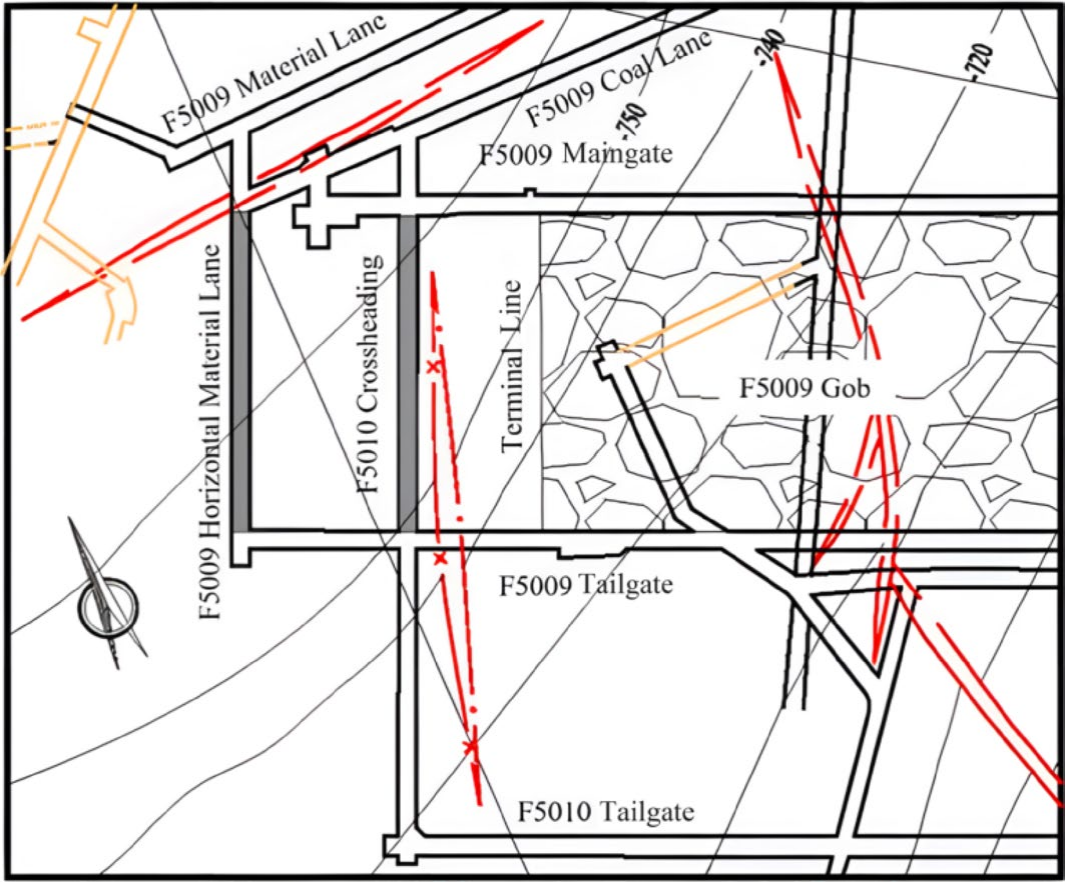
The top view of the rock burst accident area, Tangshan coal mine, China.

The Tangshan Coal Mine is located in Tangshan, China. Development commenced in 1878, and operations began in 1881, giving a mining history of 145 years. The mining area is 55.01 km^2^, and the current production is 3 Mt annually. The 5#, 8#, and 9# coal seams are currently being mined, with a burial depth of approximately 800 m. The geological structure of the coalfield is complex, and folds and faults are developed. There are five main faults in total, causing multiple anticlines and synclines in the coal seam. The rock burst occurred in the F5010 crossheading and F5009 material hauling lane. The top view of the accident area is shown in Fig. 1.

The accident occurred in the roadways of the F5009 panel. The south of the F5009 working face is the F5010 unmined panel, while the north is the F5009 roadways. The average coal thickness of the F5009 working face is 2.6 m, and its average inclination angle is 17°. At the time of the accident, the F5009 panel had already been mined out, and the hydraulic shield stayed at the mining stopping line for working face removal.

The length of the F5009 material lane is approximately 110 m, approximately 71.5 m from the mining stopping line. The elevation of the roadway is − 755.2 to − 777.6 m, and it is excavated along the roof of the 5# seam. The roadway is supported by rockbolts and steel mesh, and a U-steel canopy is used for secondary reinforcement. Another accident site is the F5010 roadway, which has a connecting alley length of 178 m and is 31 m away from the F5009 mining stopping line. The cross section of the roadway is 4.5 m × 3.0 m, connecting four tunnels: the F5010 air duct, F5009 air duct, F5009 chute, and F5009 coal-conveying roadway. The buried depth of the tunnel is between 758.6 and 775.6 m. The roadway is excavated along the roof of the 5# coal seam and is also supported by rockbolt and mesh.

**Figure 2:**
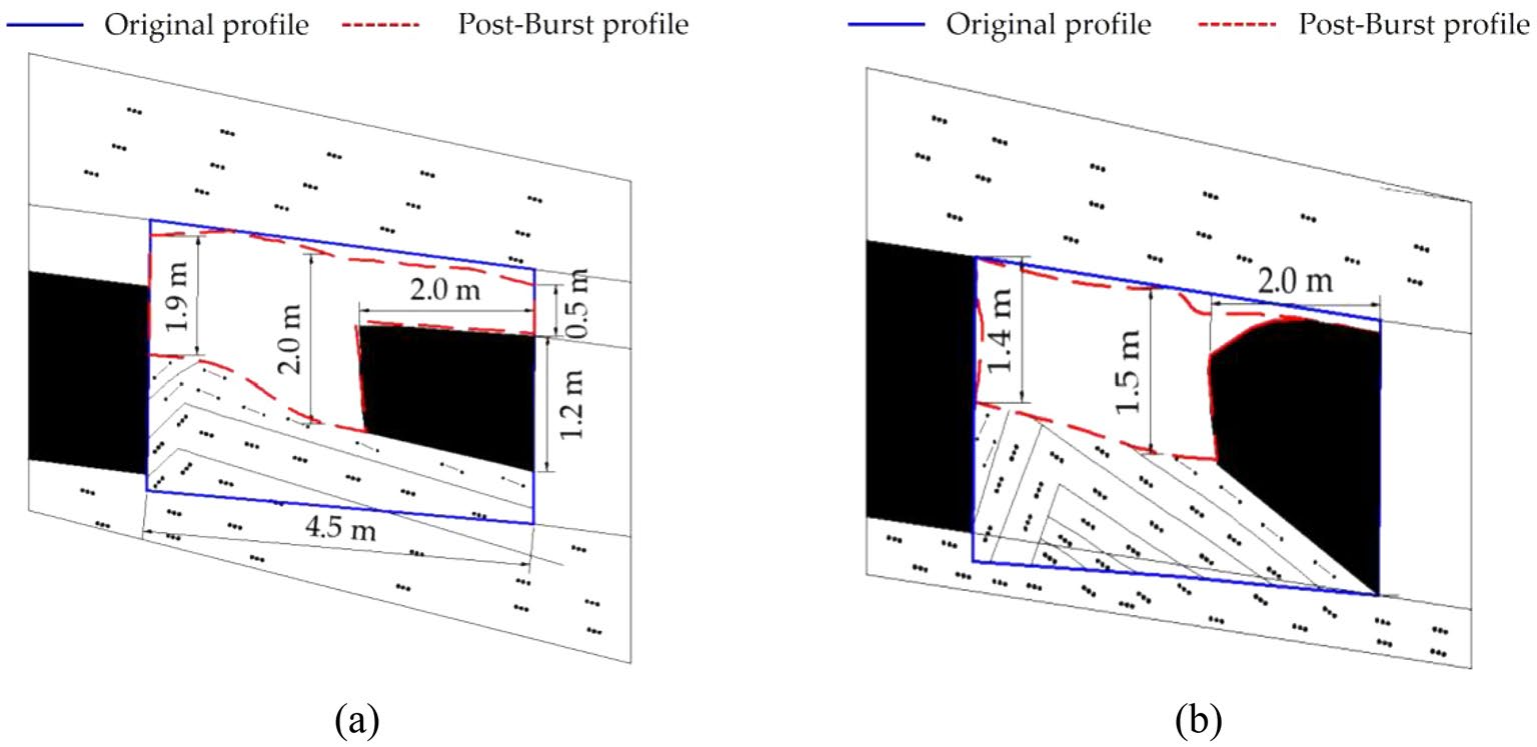
Profiles of the F5010 roadway pro- and post-rock-burst accident: (**a**) 17.6 m away from the F5009 chute and (**b**) 28 m away from the F5009 chute.

In a rock burst accident, the F5010 connecting roadway was severely damaged for a length of 80 m. After the rock burst occurred, an accident investigation was conducted on the F5010 connecting roadway, and sketches of the roadway damage were drawn, as shown in Fig. 2.

In Fig. 2a, the roadway is located 17.6 m away from the F5009 chute. After the impact, the height of the centreline of the tunnel left was 2.0 m in height, the height of the lower rib was only 0.5 m, and the coal mass on the lower rib shifted approximately 2.0 m into the tunnel. The height of the upper rib was 1.9 m, and the roof W-steel strip was apparently deformed. In Fig. 2b, the roadway is located 28 m away from the F5009 chute. The height of the central line of the tunnel is 1.5 m, and the rib coal on the lower side is offset by 2.0 m towards the tunnel, resulting in complete closure of the lower side tunnel, and a height of 1.4 m on the upper rib remains.

In summary, the impact damage in the F5010 connecting roadway is that the rib coal of the lower rib slid into the roadway as a whole, and the length of the coal body rushed into the roadway was approximately 2.0 m. There was no obvious damage to the upper rib. The roof of the roadway was not significantly damaged, nor was there large roof subsidence. The roof support was not damaged either. The floor underwent bending failure, and on the lower rib, the floor flowed into the tunnel, together with the coal mass, with a floor heave height of 1.0–1.5 m.

After the rescue and on-site investigation of the accident, the Tangshan Coal Mine stopped production for accident analysis. If the causality and process of the accident cannot be clarified or related prevention and control measures are not undertaken, then the mine may be closed. This study adopted numerical calculation methods to analyse the mechanism of the accident and to provide targeted measures.

### In situ stress measurements

The ground stress field of the Kailuan coalfield belongs to the geo-dynamic type (compression zone), but the ground stress distribution of the Tangshan Coal Mine is complex. The fault and fold structure divide the mine into several geo-structural units, and the difference in the in-situ ground stress between structural units is large.

**Figure 3:**
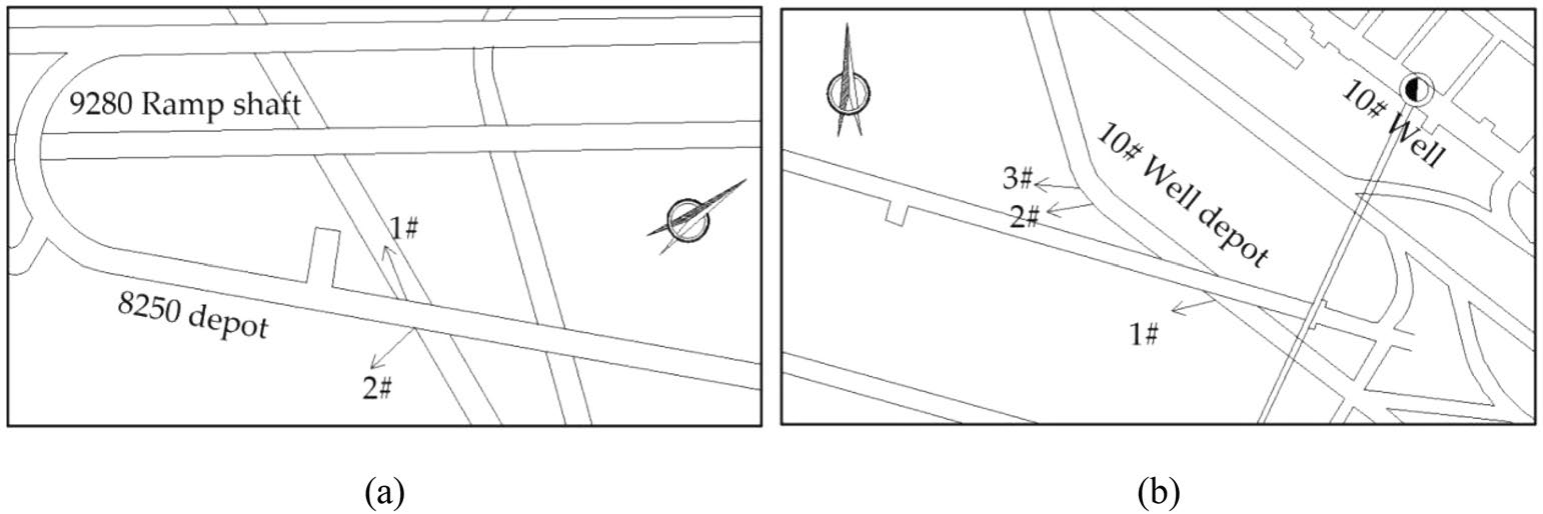
In situ stress measurement points: (**a**) 8250 depot and (**b**) #10 well depot.

In situ ground stress measurements were carried out at the 8250 depot and #10 well depot, as shown in Fig. 3. There are a total of 5 measurement points, and the results are shown in Table 1. The results show that the maximum principal stress in the measured area is in the horizontal direction, with an orientation of northeast–southwest and a size of 31.30–33.33 MPa. The intermediate principal stress is vertical stress, ranging from 20.48 to 22.09 MPa, with an average increase of 2.86 MPa per 100 m elevation (the theoretical value is 2.5 MPa). The maximum principal stress in the horizontal direction is approximately 1.5 times the vertical stress. Therefore, the accident area is identified as a high ground stress area dominated by horizontal tectonic stress.

**Table 1:** In situ stress measurement results, σ_1_, σ_2_, and σ_3_ represent the major, medium and minor primary stress, respectively.

Measurement locations	Point	Stress	Value (MPa)	Orientation (°)	Dipping (°)
8250 depot (− 800.3 m)	1	σ_1_	33.00	259.30	2.01
		σ_2_	22.09	− 18.57	− 75.60
		σ_3_	19.60	169.81	− 14.25
	2	σ_1_	33.33	264.57	0.86
		σ_2_	22.19	− 1.11	78.76
		σ_3_	21.47	174.40	11.21
#10 well depot (− 706.2 m)	1	σ_1_	31.73	255.14	2.10
		σ_2_	20.48	− 8.76	70.92
		σ_3_	19.83	164.42	18.95
	2	σ_1_	31.33	256.10	0.53
		σ_2_	21.22	− 12.39	69.72
		σ_3_	20.55	165.99	20.27
	3	σ_1_	31.30	266.90	3.49
		σ_2_	21.04	4.26	64.54
		σ_3_	20.05	175.26	25.18

### Overlying strata detection

To improve the accuracy of the calculation results, it was necessary to identify the overlying strata structure of the F5010 roadway. The roof structure of the 5# coal seam near the accident area was investigated using a YTJ-20 rock layer detection recorder. The detection borehole size was 42 mm, drilled perpendicular to the roof, and the observation depth was 18 m.

**Figure 4:**
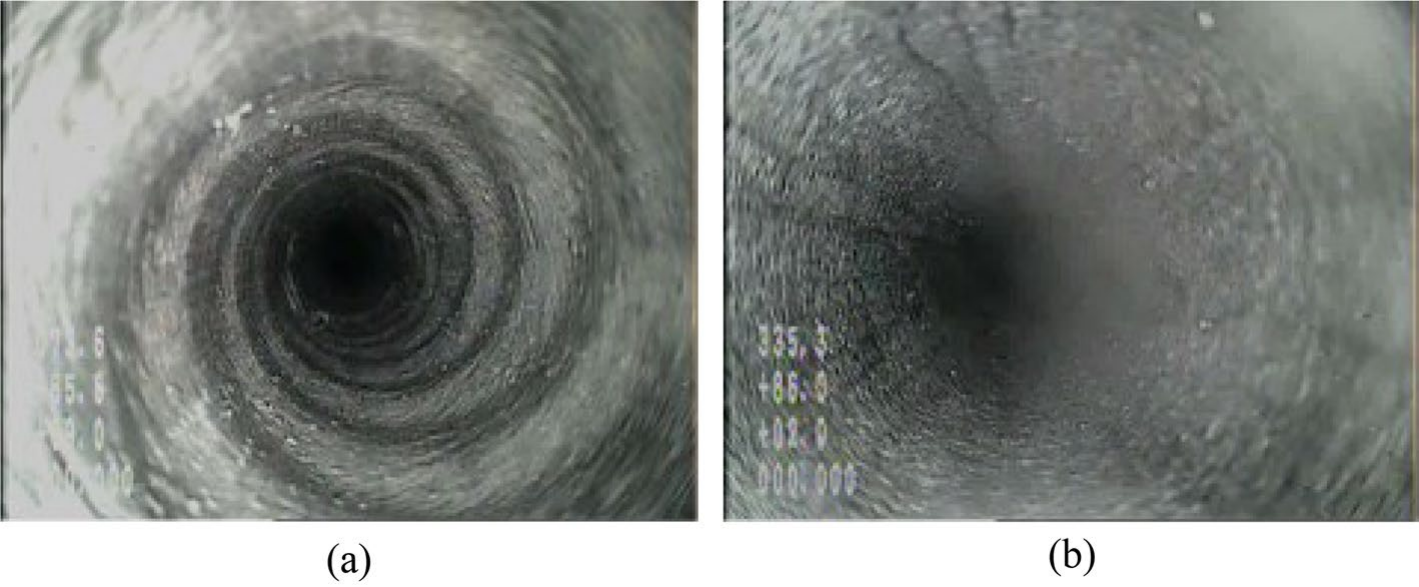
Observation images: (**a**) hole depth 2.0 m and (**b**) hole depth 10.3 m.

Figure 4 shows the images of the detection borehole. At a hole depth of approximately 2 m, black and white rocks alternated, suggesting that it was the boundary between the immediate roof and the main roof (Fig. 4a). After exceeding a depth of 2 m, the borehole wall became smooth, with no significant changes in lithology. Cracks were observed in the rock mass around a borehole depth of 10.3 m (Fig. 4b); however, the integrity was restored in the upper rock layer.

The results showed that the thickness of the immediate roof of the 5# coal seam in the detected area was approximately 2.0 m, and the thickness of the main roof exceeded 16 m. The integrity of the overlying rock layer was high; there were cracks in the local area that did not extend. It is believed that the cracks had little effect on the deformation and movement of the roof.

### Mechanical parameters testing

The roof and coal seam near the observation hole were cored with a drilling diameter of 110 mm. The rock cores in the first 2.0 m were broken in-hole. A total of 39 effective rock cores were extracted from the roof of the 5# coal seam, with a marked depth of 2.0–18.7 m. The roof core was divided into three layers, from shallow to deep: roof 1—siltstone, roof 2—medium sandstone, and roof 3—siltstone.

According to the national standard of “Methods for Determining the Physical and Mechanical Properties of Coal and Rock” GB/T 23561.7-2009^27^, the physical and mechanical parameters of the 5# coal seam and the 3 roof layers were tested; the results are shown in Table 2. The uniaxial compressive strength of sandstone in the main roof was 66.1 MPa, with an elastic modulus of 37.8 GPa, indicating that it belonged to the hard rock layer.

**Table 2:** Physical and mechanical parameters of the 5# coal seam and roof strata.

Strata	Thickness (m)	Density (kg m^−3^)	UCS (MPa)	E (GPa)	UTS (MPa)	Cohesion (MPa)	Internal frictional angle (°)
Roof-3	5.7	2567	26.1	27.3	3.8	–^1^	–^1^
Roof-2	10.9	2681	66.1	37.8	2.5	24.1	30
Roof-1	2.1	2599	22.4	10.6	2.7	15.7	24
5# seam	2.5	1389	8.3	3.0	0.67	1.8	35
Floor	5.2	2767	28.9	29.5	5.3	–^1^	–^1^

^1^Note: “–” means data not obtained.

### Calculation model

**Figure 5:**
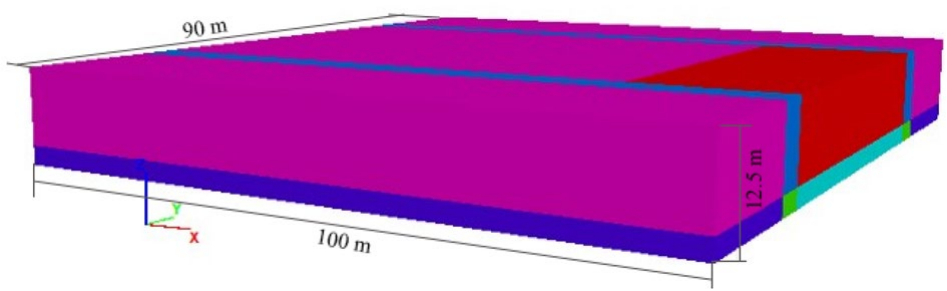
The calculation model.

To study the causality mechanism of the rock burst accident that occurred in the Tangshan Coal Mine, a 3D numerical calculation model of the F5009 working face was established using a finite difference method, as shown in Fig. 5. This calculation model mainly focused on the stress distribution of the F5009 panel and the F5010 roadway. The model size was set to 90 m in length and 100 m in width, with a thickness of 12.5 m. The length of the unmined coal in front of the stopping line of the F5009 panel was 70 m, which is the same as reality.

To obtain the stress distribution of the panel while the accident occurred, the element in the calculation model was selected as an elastic material; this approach avoided nonconvergence of the calculation caused by shear failure when using a Mohr–Coulomb material. Since rock burst is a type of sudden and violent massive failure, conventional compression shear failure that slowly occurs in the coal and rock mass was not the dominant failure mode in the disaster; therefore, this assumption was acceptable. This kind of analogy method has also been adopted by other researchers^28,29^.

In terms of the boundary conditions, the bottom of the model had displacement constraints for all directions, while other elements were free to move. Based on the in situ stress measurement results (Table 1), the initial stress could be applied. To simplify the calculation, a vertical stress was applied on the top of the model to simulate the gravity of the overlying strata.

During the development and excavation of underground coal mines, the original rock stress is disrupted, and the overburden pressure upon excavation is redistributed to surrounding rocks^30^. This leads to the vertical stress of the rib coal and coal near the working face exceeding their original stress, which is the so-called abutment pressure^31^. In theory, the abutment pressure caused by the suspended roof in front of the working face is 1.5–5.0 times the original vertical stress^32,33^. In this study, the abutment pressure was simulated by applying 2 times the vertical stress above the suspended roof in the calculation model. In the horizontal direction, based on the magnitude and direction of the original rock stress measurement in Table 1, the major principal stress was applied along the normal direction of the roadway rib, and the minor principal stress was applied along the axial direction of the roadway.

Based on the on-site roof detection results, in the assigned model, the roof had to a hard and thick uniform lithology. Based on the measurement results of the physical and mechanical parameters of the coal and rock masses in the laboratory, the rock layer attribute parameters in the numerical calculation model were determined, as shown in Table 3.

**Table 3:** Physical and mechanical parameters of coal and rock in the model.

Rock layers	Volumetric modulus (GPa)	Shear modulus (GPa)	Tensile strength (MPa)	Internal frictional angle (°)	Cohesion (MPa)	Density (kg m^−3^)
Roof	24.3	15.2	2.50	30	24.1	2681
Coal	2.8	1.5	0.67	35	1.8	1389

## Results

### The stress distribution of the panel

**Figure 6:**
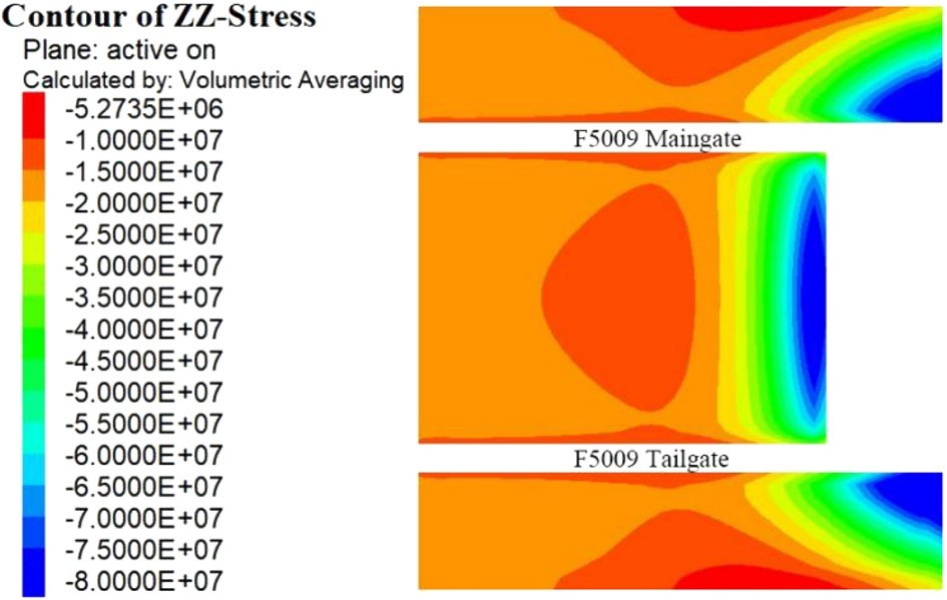
Normal stress distribution of the coal–rock interface of the F5009 panel.

Figure 6 shows the calculation results of the vertical stress at the coal–rock interface in the model. The results show that the stress of the coal body near the stopping line was at a maximum. This is in agreement with abutment pressure theory and is consistent with the fact that the 5# coal seam strength is high. From the working face forward, the vertical stress of the interface gradually decreased. In the range of approximately 27–35 m along the centreline of the working face, a low stress zone was found. The F5010 connecting roadway is located 31 m ahead of the stopping line, which is within the vertical stress relief area.

**Figure 7:**
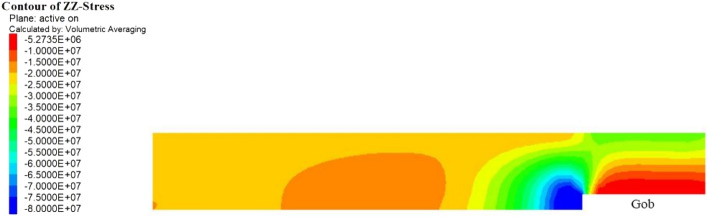
Stress distribution of the centre plane of the F5009 panel.

To further analyse the stress state of the coal and rock mass in front of the F5009 working face, the stress distribution of the centreline of the panel was plotted, as shown in Fig. 7. The coal body pressure was the highest within approximately 3 m ahead of the F5009 working face. Then, the vertical stress gradually decreased in the area from 3 to 15 m, but it was still higher than the original ground stress, indicating that it was still within the area of compressive abutment pressure. The stress 15–18 m in front of the working face was restored to the in-situ stress. Interestingly, the vertical stress 18–36 m ahead of the working face was lower than the original ground stress. Therefore, it was suggested that the decrease in normal stress of the roof–coal interface in front of the working face was the cause of the rock burst disaster.

The stress concentration coefficient of the abutment pressure is related to the structure and mechanical properties of the overlying strata. Under the hard-roof conditions of the 5# seam in the Tangshan Coal Mine, a long cantilever beam was formed by the suspended main roof, as it was not easy to collapse. As a result, the vertical stress of the overlying strata on the suspended roof was also borne by the coal body near the working face. As the F5009 working face was mined to the stopping line, the abutment pressure generated by the suspended roof caused the coal body near the working face to be in a high-pressure state. The numerical calculation results also showed this consequence.

### The causality mechanism of the disaster

The 5# coal seam of the Tangshan Coal Mine is a hard coal seam with a high bearing capacity, especially under confinement pressure. Engineering experience shows that the elastic–plastic deformation boundary within the rib of the roadway driven in a hard coal seam is generally less than a depth of 2 m; that is, from approximately 2 m in front of the F5009 working face, the horizontal ground stress of the coal body is close to its original stress. Therefore, it can be concluded that the coal body 2 m ahead of the stop mining line is in a high confining pressure state. Under confining pressure, the bearing capacity of the 5# coal body significantly increased. A large number of true triaxial test studies have been conducted^33,34^. Shen et al.^35^ showed that the strength of coal increases as a power function with the confining pressure on the sample. Therefore, it is believed that the majority of the coal in the F5009 working face was located in the elastic deformation state.

The coal body 2 m ahead of the working face was in elastic deformation, so its strain had a linear relationship with the abutment pressure. The vertical compressive displacement is only related to the coal seam stress, seam elastic modulus, and mining height. Therefore, the seam had the characteristics of high vertical stress but low displacement; that is, the coal body near the working face had a high compressive stiffness. This feature is also consistent with the calculated results.

**Figure 8:**
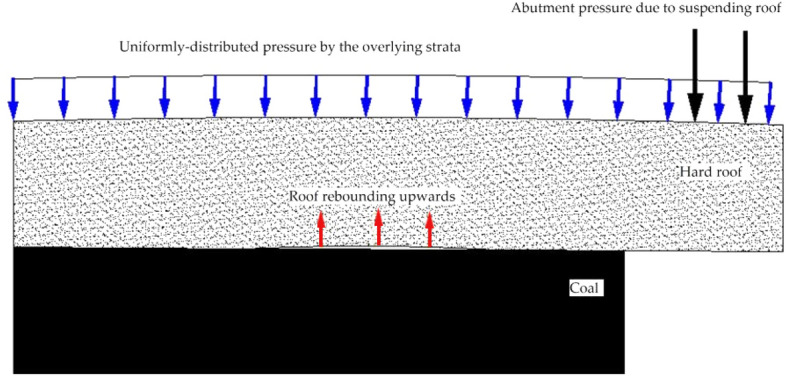
Upward rebounding of the roof in front of the working face.

The coal body near the working face had a high compressive stiffness, and due to the high abutment pressure generated by the suspended hard roof, the interface between the roof and the coal seam in front of the working face rebounded upwards, which is the pressure relief area shown in Figs. 6 and 7. Under the conditions of the hard roof and hard coal seam, the upward rebounding of the roof in front of the working face resulted in pressure relief at the roof–coal interface, as shown in Fig. 8.

In Fig. 8, the short blue arrow represents the gravity of the overlying strata. The suspended roof generated by the mining process formed a cantilever beam, and the abutment pressure is represented by the black arrows. The coal body with high bearing capacity near the working face had high compressive stiffness, which meant that the vertical displacement of the coal seam was small, inevitably causing the roof in front of the work to rebound upwards. Overall, the working face formed a structure that was similar to a seesaw. The coal body near the working face was the fulcrum, and the roof near the fulcrum was the pressure plate. The abutment pressure behind the working face acted on the suspended roof, causing the roof in front of the working face to move upwards.

The F5010 roadway is parallel to the F5009 working face. Since the F5010 roadway is in the pressure relief area, the clamping force of the roof and floor on the rib coal was weakened. As a result, the rib coal rushed into the roadway under the action of horizontal ground stress, which was the direct cause of the accident. Notably, the accident site showed that the floor heave of the F5010 roadway was large; however, the floor did not actively induce the impact. Instead, the floor heave was caused by the rib coal moving into the roadway.

## Discussion

### Comparison with similar studies

A field research method was adopted, and the geological and mining conditions of the impact site were detected and analysed. Then, a numerical model was established to restore the stress state of the working face at the time of the accident. Based on the calculation results, a mechanical model of the occurrence mechanism of this type of rock burst was obtained, which is highly innovative.

The new model of the mechanism of rock burst in tunnels shows that with high abutment pressure and the high compressive stiffness of the coal body in front of the working face, the roof in the front roof of the working face rebounded upwards. This lead to a decrease in the clamping force of the roof and floor acting on the coal body and a subsequent decrease in the sliding resistance between the roof and floor with the coal body, causing the horizontal stress to overcome the sliding resistance. The rib coal mass slipped and flowed into the roadway, facilitating a rock burst.

### Limitations and further research directions

This study is a case-based on-site study, and the results are only applicable to roadway rock burst under hard roof and hard coal seam geological conditions. This model is not applicable to the coal bump that occurs at the working face, during tunnel excavation, or in the form of crater impact. Moreover, due to the permanent closure of the accident tunnel, no drilling analysis was conducted at the accident tunnel to further validate the mechanical model proposed in this paper.

With the development of deep resource extraction, rock burst has become increasingly frequent and severe. In response to the new theory of roadway rock burst established in this study, it is necessary to accumulate data in the future and establish more detailed quantitative theoretical analysis methods to reduce disaster risks. From a technical perspective, the author believes that in the future, simpler and more reliable monitoring technologies and equipment should be developed, a systematic system for predicting rock burst should be established, and the relationship between the abutment pressure of the rear suspended roof and the rebound data of the roof in front of the work face should be automatically analysed, providing a simple and reliable decision-making basis for field engineers.

### Corresponding prevention measures

The Tangshan Coal Mine is located in the Kaiping syncline axis of the Kailuan coalfield. The accident was the first rock burst disaster in its 144-year mining history. If the Tangshan Coal Mine cannot ensure mining safety in deep resource mining, then it will be closed. Additionally, other subordinate coal mines of Kailuan (Group) Co., Ltd., have rock burst hazards due to similar geological and mining conditions. Therefore, it is of great significance to conduct basic theoretical research and establish a targeted prevention and control technology system.

Numerical studies have shown that the rock burst disaster in the Tangshan Coal Mine was mainly induced by the hard roof and hard coal seam. Therefore, two rock burst prevention measures were introduced for the Tangshan Coal Mine to eliminate rock burst disasters.

### Large-diameter pressure relief borehole drilled in coal seams

**Figure 9:**
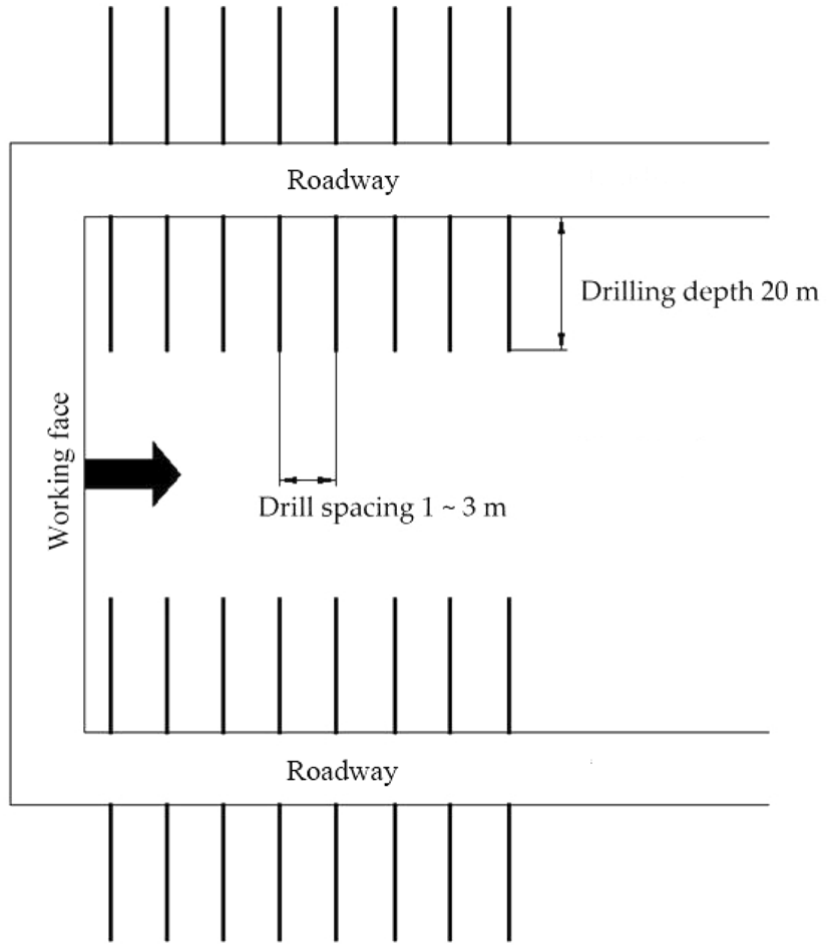
Layout of large-diameter pressure relief boreholes.

For the coal seams with high bearing capacity, large-diameter pressure relief boreholes were drilled within the rib coal to prevent the hard coal in front of the working face from forming a “fulcrum” structure. The boreholes drilled in both ribs had a diameter greater than 100 mm and a depth of 20 m. The holes were 1.0–1.5 m away from the floor, with an interval of 1–3 m (Fig. 9). The drilling depth could be adjusted according to site conditions.

### Deep blasting of roof.

**Figure 10:**
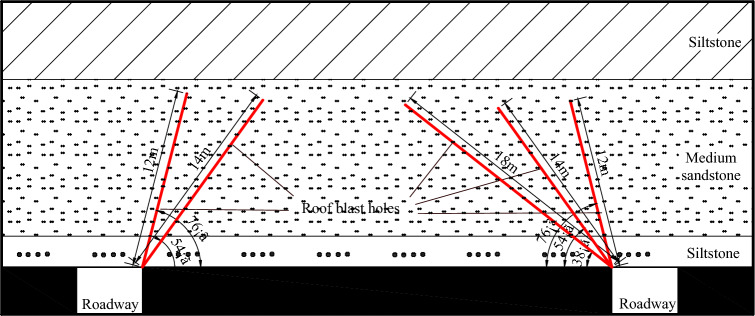
Layout for the deep blasting of the roof.

It is difficult for a hard roof to collapse, and there is a tendency to form a “pressure plate”. Thus, a deep hole blasting method was proposed to weaken the roof. Hydraulic drilling machines were used for the operation, with a drilling diameter of greater than 72 mm. A group of drilling holes was arranged 10–15 m in the mining roadway, with 3–5 blast holes in each group (Fig. 10). According to site conditions, the parameters of the blasting hole interval, angle, and depth can be adjusted. This method destroys the integrity of the roof to eliminate the formation of a “pressure plate”.

Based on the geological characteristics of the hard roof, hard coal seam, and high horizontal ground stress in the Tangshan Coal Mine and based on the calculation results of the occurrence mechanism of the rock burst accident, rock burst prevention measures of large-diameter drilling in the coal seam and roof blasting were adopted. After implementation, the Tangshan Coal Mine safely mined its Y394, 0250, 0291, and Y251 panels, producing 4.45 Mt of raw coal, resulting in good social and economic benefits. The research results of this study also provide a reference for safe and efficient mining under similar conditions in the Kailuan coalfield.

## Conclusions

This study used numerical methods to calculate and analyse the rock burst accident that occurred in the Tangshan Coal Mine and to establish corresponding prevention and control measures based on the mechanism of rock burst occurrence. The conclusions are summarized as follows:

1. The in situ ground stress measurement showed that the horizontal ground pressure coefficient of the Tangshan Coal Mine was 1.5, indicating that the in situ ground stress of the coal mine was dominated by tectonic stress. The results of the roof detection and physical and mechanical experiments of coal and rock showed that the F5009 working face is part of a hard coal seam, with a hard and thick sandstone roof.
2. A numerical calculation model for the F5009 working face was established based on the site’s geological conditions. The calculation results showed that the coal body near the stopping line of the F5009 working face was in a high stress zone, and the influence range of abutment pressure was approximately 15 m. There was a pressure relief zone at 18–36 m in front of the working face, where the clamping force of the roof and floor on the coal body was reduced, causing the rib coal to overcome the frictional resistance of the roof and floor and rush into the roadway, resulting in the rock burst accident.
3. Based on the numerical calculation results and analysis of the occurrence mechanism of the accident, targeted prevention measures of large-diameter borehole drilling of rib coal and roof blasting were proposed. Engineering practice showed that these measures had good effects.

Based on the characteristics of rock burst accidents in the Tangshan Coal Mine, in this study, numerical methods were used to analyse the accident mechanism and establish targeted prevention and control measures. The results of this study provide a reference for safe and efficient mining under similar conditions in the Kailuan coalfield.

## Data Availability

The original contributions presented in the study are included in the article/supplementary material. Further inquiries can be directed to the corresponding author. The data used to support the findings of this research are included within the paper.
